# Emotional behavioral outcomes of children with unilateral and mild hearing loss

**DOI:** 10.3389/fped.2023.1209736

**Published:** 2023-10-04

**Authors:** Jun Jean Ong, Libby Smith, Daisy A. Shepherd, Jessica Xu, Gehan Roberts, Valerie Sung

**Affiliations:** ^1^ Centre for Community Child Health, The Royal Children’s Hospital, Melbourne, VIC, Australia; ^2^ Murdoch Children’s Research Institute, Melbourne, VIC, Australia; ^3^ Department of Paediatrics, School of Medicine, International Medical University, Kuala Lumpur, Malaysia; ^4^ Department of Paediatrics, The University of Melbourne, Melbourne, VIC, Australia

**Keywords:** unilateral, mild, hearing loss, emotional behavioral difficulties, deaf or hard of hearing, children

## Abstract

**Background:**

Deaf and hard-of hearing (DHH) children often experience emotional/behavioral difficulties. The impact of unilateral/mild hearing loss (HL) on children's emotion and behavior are unclear. We aimed to describe emotional/behavioral, health related quality-of-life (HRQoL) and parent psychological distress outcomes of school-age children with unilateral/mild HL, compared to children with moderate to profound HL, and in relation to population norms; and identify predictive factors of emotional/behavioral difficulties.

**Methods:**

Data of 339 DHH children, 5–12 years, enrolled in the Victorian Childhood Hearing Longitudinal Databank (VicCHILD), which include demographics, early development, medical/audiological characteristics and parent rated questionnaires of emotion/behavior, HRQoL and parental psychological distress collected at various stages of child's life were analyzed. We used Cohen's d to investigate the outcomes by measuring the mean score differences of both groups with published norms and logistic regression to analyze the factors predictive of emotional/behavioral difficulties.

**Results:**

The proportion of children with unilateral/mild HL and moderate to profound HL who experienced emotional/behavioral difficulties was similar (18.3% vs. 20.6%), with hyperactivity and poor prosocial behavior reported as the predominant symptoms in both groups. Mean emotional/behavioral scores of both groups were comparable and substantially higher than normative population scores. This was also the case for HRQoL and levels of parent distress. Among children with unilateral/mild HL, additional health needs were the strongest predictive factor, demonstrating an approximately 1.7-fold increase in odds of emotional/behavioral difficulties (OR = 1.67; 95% CI 1.29–2.17, *p* < 0.001) with every additional health need. Early developmental concerns, other than communication milestone and attending mainstream schoolshowed weaker evidence of association.

**Conclusion:**

Children with unilateral/mild HL were just as likely as those with moderate to profound HL to experience more emotional/behavioral difficulties, poorer HRQoL and higher parental distress scores compared to population norms. Our findings justify the provision of early intervention, support and medical services for all DHH children to identify those at risk of poorer outcomes.

## Introduction

Deaf and hard-of hearing (DHH) children often experience emotional and behavioral difficulties (1, 2). Previous studies, which mainly include children with moderate to profound hearing loss (HL) have reported high prevalence of externalizing and internalizing behavioral symptoms compared to normal hearing population (2). In a review by Stevenson et al. (2015), peer problems were rated by both parents and teachers as the predominant emotional/behavioral symptoms, whereas a recent longitudinal study showed that hyperactivity/inattention symptoms were most reported by parents and low prosocial behavior by teachers (2, 3). Deficits in socio-emotional regulation due to delayed executive function and social cognitive development are hypothesized to be reasons DHH children are more vulnerable to emotional/behavioral problems (4). Poor social skills and low prosocial behavior are equally reported despite improved language development due to persistent pragmatic developmental challenges (5). However, more recent studies have described minimal differences in emotional/behavioral mean scores among DHH children compared to normative data (2, 6). The narrowing of the gap in emotional/behavioral outcomes are likely contributed by the implementation of universal newborn hearing screening (UNHS) and the advancement of audiological intervention, speech-language therapy and counselling services in the past decade. Nevertheless, emotional/behavioral outcomes remain highly variable with a large proportion of DHH children continuing to experience difficulties despite earlier access to intervention and improvement of language and academic performances (7). Ongoing research to investigate possible contributing factors is much needed.

Language and communication development are among the most well-studied predictors of emotional/behavioral outcomes in DHH children (6, 8, 9). The severity of emotional/behavioral problems are influenced by the level of language abilities, with good receptive language and communication skills associated with lower risk of emotional/behavioral difficulties (8, 9). Aside from language abilities, additional health needs and nonverbal cognitive ability were significant factors identified among DHH children at 3 and 5 years of age (6, 10). Given that approximately two thirds of DHH children are reported to have an additional disability that could impact their education or development, Wiley et al. (2011) proposed the need for interdisciplinary medical evaluation for all DHH children (11). Studies have also shown that early detection of hearing loss and early access to intervention were associated with favorable academic and language performance (2, 8). However, degree/laterality of HL have not been shown to influence outcomes in several studies, with Wake et al. (2004) reporting that DHH children have poorer psychosocial, quality of life and language outcomes, irrespective of the severity of HL (1, 7, 12). In addition, Carew et al. (2018) reported poorer expressive language skills in children with mild HL compared to population means, despite early detection through the well-established UNHS (13).

Increasing detection of children with milder HL through UNHS and the awareness of their challenges have led to a rise in research interest exploring outcomes of unilateral/mild HL (1, 14, 15). Studies suggest children with unilateral/mild HL score lower in academic tests, are more likely to fail at least one grade and are delayed in various developmental abilities compared to hearing peers (16–18). They experience more emotional/behavioral difficulties than their peers with one fifth of children with unilateral HL reported by their teachers to have behavioral problems and requiring classroom accommodations (16, 17). Studies of children with unilateral/mild HL published after the implementation of UNHS continue to report high socio-emotional problems despite early diagnosis (13). A 3-year follow-up longitudinal study of children with unilateral HL described high prevalence of behavioral problems that improved with intervention; however, 10% or more continue to have problems with inattention, externalizing and internalizing symptoms (19). Porter et al. (2013) showed that differences in academic performance between unilateral/mild HL and hearing children were not apparent, but greater attention difficulties in the classroom were identified among children with unilateral/mild HL (20). Le Clercq et al. (2020) further emphasized the association between emotional/behavioral outcomes and hearing threshold, with higher inattention and social problems among children with slight to mild hearing loss (21). However, the effects of unilateral/mild HL on emotional/behavioral outcomes remain inconclusive as available studies also showed contradicting results, reporting no additional behavior problems compared to typical hearing children (22, 23).

As not all DHH children experience emotional/behavioral difficulties, recognition of predictive factors is imperative to identify those at high risk of poorer outcome. Factors associated with emotional/behavioral outcomes among children with moderate-profound bilateral HL are well explored, but likely differ from children with unilateral/mild HL due to differences in their experiences and access to sound. Possible factors such as lower maternal education, later age of amplification and intervention were suggested to be associated with poorer outcomes among children with unilateral/mild HL (19, 20, 24). However, evidence is scarce and the additional benefits of audiological intervention on emotional/behavioral outcomes among children with unilateral/mild HL remain uncertain.

The study analyzed data of families enrolled during the first 10 years of the Victorian Childhood Hearing Longitudinal Databank (VicCHILD) which recruited children with permanent HL of any degree and laterality (25). Information from a considerably large sample of children with unilateral/mild HL were able to be included in the study to address the following research questions:

1. What are the emotional/behavioral, health related quality-of-life (HRQOL) and parent psychological distress outcomes of children with unilateral/mild HL compared to children with moderate to profound HL and the normal hearing population?
2. Which factors are predictive of emotional/behavioral difficulties among children with unilateral/mild HL?

## Material and methods

### Participants

Our cross-sectional study included DHH children enrolled during the first 10 years of VicCHILD (between 2012 and 2022), and whose families completed a survey including emotional/behavioral outcomes at age 5–12 years. VicCHILD is a statewide population-based longitudinal databank open to every child with permanent hearing loss in Victoria, Australia (25). The majority of families are recruited through the statewide UNHS program, the Victorian Infant Hearing Screening Program (VIHSP) which screens more than 99% of newborns in Victoria. Families of DDH children attending the Royal Children's Hospital Caring for Hearing in Children Clinic are also invited to participate. Information and assessments are collected at enrolment and various stages of the child's life course, with the details of methodology described elsewhere (25). Recruitment and assessments at different age timepoints are still ongoing. This study described data on emotional/behavioral outcomes, assessed during primary school-age (5–12 years) and related information collected at different stages. The study has ethics approval from the Royal Children's Hospital Human Research Ethics Committee (approval number 31081).

### Procedure

Parents provided sociodemographic, audiological and medical information about their child during enrolment and subsequent stages of assessment. Audiological data from the time of diagnosis was provided by the VIHSP. Where possible, updated audiological data was obtained at the time emotional/behavioral outcomes were collected, either from Hearing Australia, a service provider throughout Australia tasked with providing monitoring and rehabilitation services for deaf/hard of hearing children, or from the Caring for Hearing in Children Clinic, a pediatric service based at the Royal Children's Hospital. Audiological data included information about the child's type of HL, degree/laterality of HL, use of hearing devices at the time of assessment (or unaided) and age of first fitting, where available. Degree of hearing loss was classified using decibel ranges used by the national provider of hearing amplification, Hearing Australia (26): mild (21–40 dB), moderate (41–60 dB), severe (61–90 dB) and profound (>90 dB). We grouped the children according to the degree/laterality of HL: unilateral/mild HL vs. moderate-profound HL. Children with unilateral HL have mild to profound HL in one ear (≥20 dB) and normal hearing in the contralateral ear (<20 dB). Children with mild HL have mild HL (21–40 dB) in the better hearing ear. Children with moderate-profound HL have at least moderate HL (≥ 40 dB) in the better ear.

We collected data of children's developmental profile and additional health needs during enrolment. We measured early developmental profile using the Ages and Stages Questionnaire (ASQ),a brief parental questionnaire of a child's current skills and development from 1 to 66 months of age (27). Parents answered 6 questions “yes”, “sometimes” or “not yet” in each of 5 domains of development: communication, gross motor, fine motor, problem-solving skills, and personal social skills based on what their child is able to do. Each answer was scored and the sum scores for each domain were calculated. Sum scores below cut-off, defined as 2 standard deviations (SD) below the mean was considered a positive early developmental concern. We only included early developmental profile assessed during the first 36 months of age for analysis. Information of additional health needs were based on parents' selection from a comprehensive list of health conditions, comprising of conditions related to neurodevelopmental, genetic and neurological disorders, malignancy, allergy, visual impairment and other chronic disorders.

We later collected outcome measures on child's emotion/behavior, quality of life and parental well-being during early school years, assessed using standardized parent rated questionnaires. The types of information and the timepoints at which data were collected from each participant child and family are described in detail elsewhere (25).

### Outcome measures

#### Emotional behavioral outcome

The Strengths and Difficulties Questionnaire (SDQ) is a 25-item parent-rated screening measure designed to identify emotional/behavioral difficulties in children (28). The instrument comprises of five subscales: conduct problems, hyperactivity, emotional symptoms, peer problems and prosocial behavior with each subscale containing 5 items. Each item is rated on a 3-point response scale from 0= “not true” to 1= “somewhat true” and 2= “certainly true”. Higher total scores for the first four subscales and “total difficulties” score which is the sum of the first four subscales (excluding prosocial behavior) reflect difficulties, while higher scores for the prosocial subscale reflect strength. The cut-off scores for “abnormal” category corresponds to the 90th (10th for prosocial subscale) percentiles, therefore total difficulties scores falling in the top 10% of the normative distribution is indicative of clinically significant emotional/behavioral difficulties (see Supplementary Table S2). Different cut-off scores and mean scores from Australian normative data are available for children 4–6 years and 7–12 years (29, 30).

#### Health related quality of life outcome (HRQoL)

The Pediatric Quality of Life Inventory version 4.0 (Peds-QL), parent completed version was used to measure HRQoL of the child (31). The inventory comprises 23 items from four domains: physical health, emotional functioning, social functioning and school functioning and each item is rated on a 5-point Likert scale. Items are scored from 0 to 100, with higher scores indicating better HRQoL. The total scores are the mean score of the sum of all domains and the psychosocial mean score represents sum of emotional, social, and school functioning domains.

#### Parent psychological distress outcome

The Kessler Psychological Distress Scale (K6) is a 6-item self-report measure of psychological distress for adults (32). Parents indicate how often they experienced feeling sad, nervous, hopeless, restless, that everything was an effort and worthless during the past 30 days, using a 5-point Likert scale. Scores above clinical cut-off point indicate significant psychological distress.

#### Statistical analysis

The data was analyzed using Microsoft Excel and SPSS Statistic Package 26. We summarized participant characteristics for each DHH groups using means [with standard deviations (SD)] for continuous variables, medians [with interquartile ranges (IQR)] if not normally distributed and counts (with proportions) for categorical variables. Normality analysis showed that data of outcomes measures were slightly skewed and not normally distributed. For the first research question, we calculated the standardized mean difference using Cohen's d effect size to compare emotional/behavioral outcomes of both groups with published Australian norms according to age; 4–6 years and 7–17 years. Although outcomes were not normally distributed, mean scores were used for analysis as available normative population data for comparison were described in mean (SD). Same method of statistical analysis was used to compare HRQoL and parent distress outcomes in both groups compared to population norms. The outcomes (means and SD) of both DHH groups were also described and compared. Spearman's rank correlation was used to estimate correlations between continuous outcomes to describe the general observed patterns in our sample. For the second research question, we used univariable logistic regression to estimate associations between key predictors (separately) with emotional/behavioral difficulties among children with unilateral/mild HL. These predictors include categorical factors (gender, age groups (5–6 and 7–12years), hearing laterality, type of HL, unaided or aided hearing, using speech or other communication mode, attending mainstream or special/other schools and presence of early developmental concerns) and continuous factors (additional health needs, hearing devices first fitting age). Variables with multiple categories (communication mode, type of HL and school) were dichotomized for analysis due to the small numbers in several subgroups.

## Results

Between 2012 and 2022, a total of 1202 DHH children were enrolled in VicCHILD. Of these, 834 DHH children had turned age 5–12 years old at the time of data analysis in late 2022, of which 339 families had completed the survey that included the SDQ as a measure of emotion/behavior. Of those who completed the SDQ (339 families), 186 families had completed the ASQ at 36 months or younger; of these 100 had unilateral/mild HLand 86 had moderate-profound HL, 337 completed the Peds-QL and 246 completed the K6. Table 1 shows the participant demographic, audiological and medical characteristics. The study sample included a total of 339 children aged 5–12 years old, consisting of 169 children with unilateral/mild HL (49.9%) and 170 children with moderate-profound HL (50.1%). The characteristics of non-participants are summarized in Supplementary Table S1. Non-participating families were from slightly more disadvantaged areas and less likely to report use of English as a primary language at home compared to study participants. Otherwise, the groups were similar in demographic and audiological details, including maternal education and degree of HL.

**Table 1:** Participant characteristics broken down by hearing loss group.

	Unilateral/Mild HL	Moderate-profound HL
	*N* = 169	*N* = 170
Child Characteristics		
Age at diagnosis of HL (months) - mean (SD)	2.1 (4.6)	2.6 (8.6)
Age at SDQ completion (years) - mean (SD)	8.0 (2.2)	8.4 (2.3)
Age group at SDQ completion (years)—*n* (%)		
5–6 years	52 (30.8)	42 (24.7)
7–12 years	117 (69.2)	128 (75.3)
Gender, male—*n* (%)	90 (53.3)	93 (54.7)
Family characteristics; *n* (%)		
Maternal education		
Year 11 or less	10 (5.9)	20 (11.8)
Year 12	51 (30.2)	41 (24.1)
Tertiary or postgraduate	71 (42.0)	58 (34.1)
Unreported	37 (21.9)	51 (30.0)
SEIFA disadvantage index^a^- mean (SD)	1,013.9 (71.1)	1,012.8 (65.3)
Family history of HL	17 (10.1)	10 (5.9)
English as primary language at home	138 (81.7)	126 (74.1)
Audiological/Medical characteristics, *n* (%)		
Degree of HL		
Unilateral-mild^b^	14 (8.3)	
Unilateral-moderate^b^	19 (11.2)	
Unilateral-severe^b^	16 (9.5)	
Unilateral-profound^b^	37 (21.9)	
Unavailable	10 (5.9)	
Bilateral mild^c^	73 (43.2)	
Bilateral-moderate^c^		63 (37.1)
Bilateral-severe^c^		32 (18.8)
Bilateral-profound^c^		49 (28.8)
Unavailable		26 (15.3)
Hearing device		
Unaided	66 (39.1)	11 (6.5)
Hearing aid/s only	66 (39.1)	57 (33.5)
Cochlear implant	6 (3.5)	80 (47.1)
Unreported	31 (18.3)	22 (12.9)
Age hearing aid first fitting		
Median (IQR), months	21.0 (43.0)	4.1 (9.0)
≤36 months, *n* (%)^d^	70 (68.6)	141 (94.0)
Age of cochlear implantation		
Median (IQR), months	35.5 (32.5)	18.0 (21.0)
≤36 months, *n* (%)^d^	3 (50.0)	62 (77.5)
Hearing loss types		
Sensorineural	123 (72.8)	130 (76.5)
Auditory neuropathy	7 (4.2)	17 (10.0)
Mixed	21 (12.4)	20 (11.7)
Permanent conductive	10 (5.9)	1 (0.6)
Atresia	8 (4.7)	2 (1.2)
Communication mode		
Speech only	143 (84.6)	114 (67.1)
Sign language (Auslan) only	0	6 (3.5)
Simultaneous sign and speech	3 (1.8)	13 (7.6)
Non-verbal/Key word signing/ gestures.	1 (0.6)	10 (5.9)
Unreported	22 (13.0)	27 (15.9)
School		
Mainstream ± special unit	139 (82.2)	118 (69.4)
School for DHH	2 (1.2)	15 (8.8)
Special school for children with disabilities	5 (3.0)	20 (11.8)
Others	5 (3.0)	3 (1.8)
Unreported	18 (10.6)	14 (8.2)
Additional health needs	131 (77.5)	124 (72.9)
Early developmental concerns ≤36 months^d^		
Communication	27 (27.0)	47 (54.7)
Gross motor	16 (16.0)	23 (26.7)
Fine motor	13 (13.0)	24 (27.9)
Problem solving	14 (14.0)	25 (29.1)
Social/Adaptive skill	13 (13.0)	21 (24.4)

^a^ Socio-Economic Indexes for Areas (SEIFA) Index of Relative Socio-Economic Disadvantage (national mean 1,000, SD 100, with higher values representing less disadvantage).

^b^ Degree of HL in the worse ear.

^c^ Degree of HL in the better hearing ear.

^d^ Total number may vary from total participants.

Compared to children with moderate to profound HL, children with unilateral/mild HL were first fitted with hearing aid at an older age (median (IQR) 21.0 (43.0) months vs. 4.1 (9.0) months) with fewer children fitted before 36 months of age (68.6% vs. 94.0%). A higher proportion of children with unilateral/mild HL were also unaided with hearing devices (39.1% vs. 6.5%), using speech as main communication mode (84.6% vs. 67.1%) and attending mainstream school (82.2% vs. 69.4%). However, around half of parents of children with moderate to profound HL reported early communication developmental concerns, double in proportion compared to children with unilateral/mild HL. Approximately one fourth of parents with moderate-profound HL children also reported early developmental concerns in all domains aside from communication milestone, higher than children with unilateral/mild HL (Table 1).

### Aim 1: outcomes of children with unilateral/mild HL compared to children with moderate-profound HLand population norms

**Figure 1:**
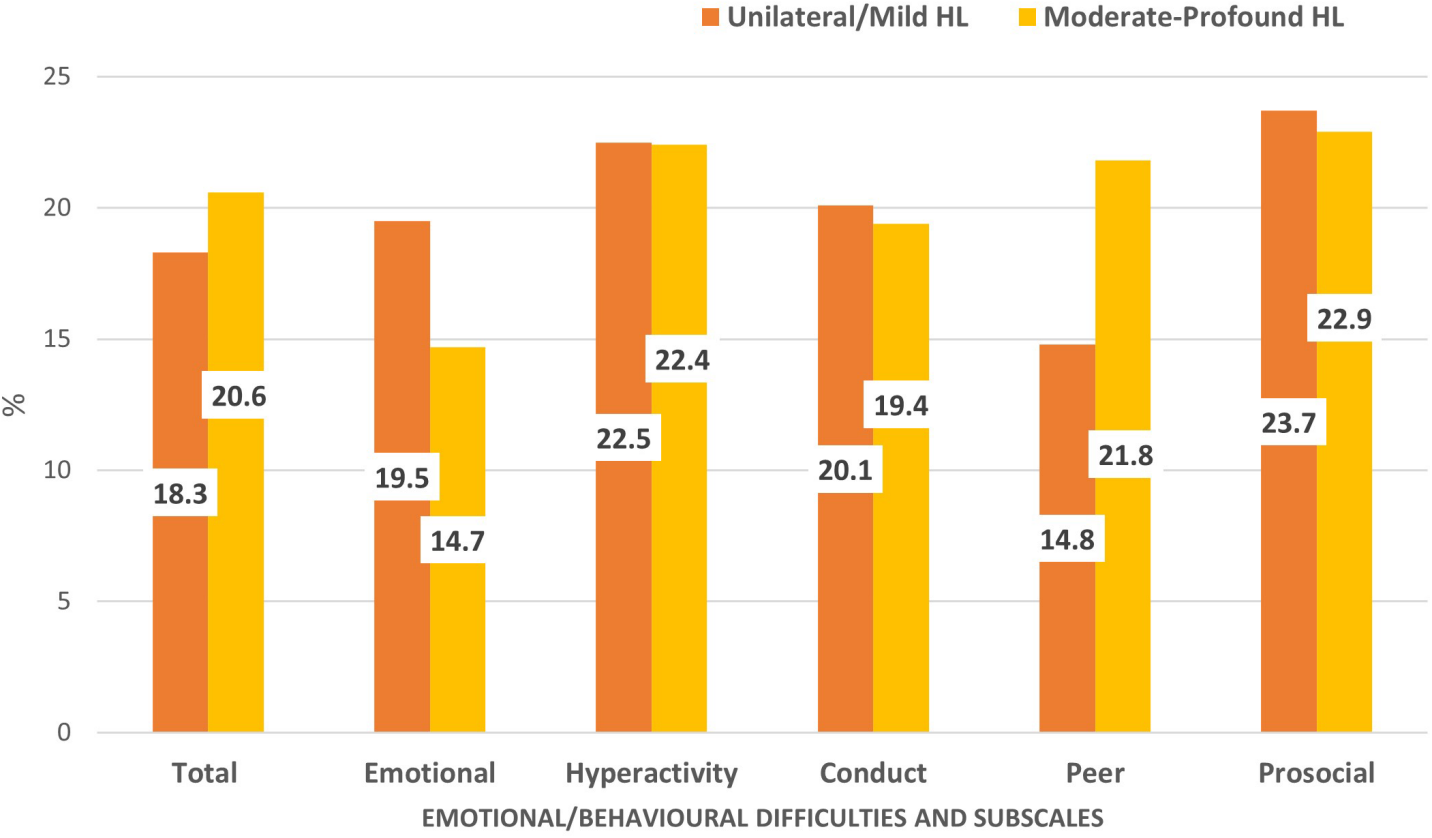
Proportion of children with unilateral/mild HL (*n* = 169) and moderate-profound HL (*n* = 170) with emotional/ behavioral difficulties and subscales scores above cut-off. * Cut-off scores are based on Australia normative data, retrieved from Kremer et al, 2015 and Mellow D., 2005.

Figure 1 shows the prevalence of emotional/behavioral difficulties of children with unilateral/mild HL and moderate to profound HL in our sample. A similar proportion of children from both groups experienced emotional/behavioral difficulties (18.3% vs. 20.6%), with hyperactivity and poor prosocial behavior, the most frequent symptoms reported. More children with unilateral/mild HL reported emotional symptoms (19.5% vs. 14.7%) while children with moderate to profound HL more often reported peer problems (14.8% vs. 21.8%).

**Figure 2:**
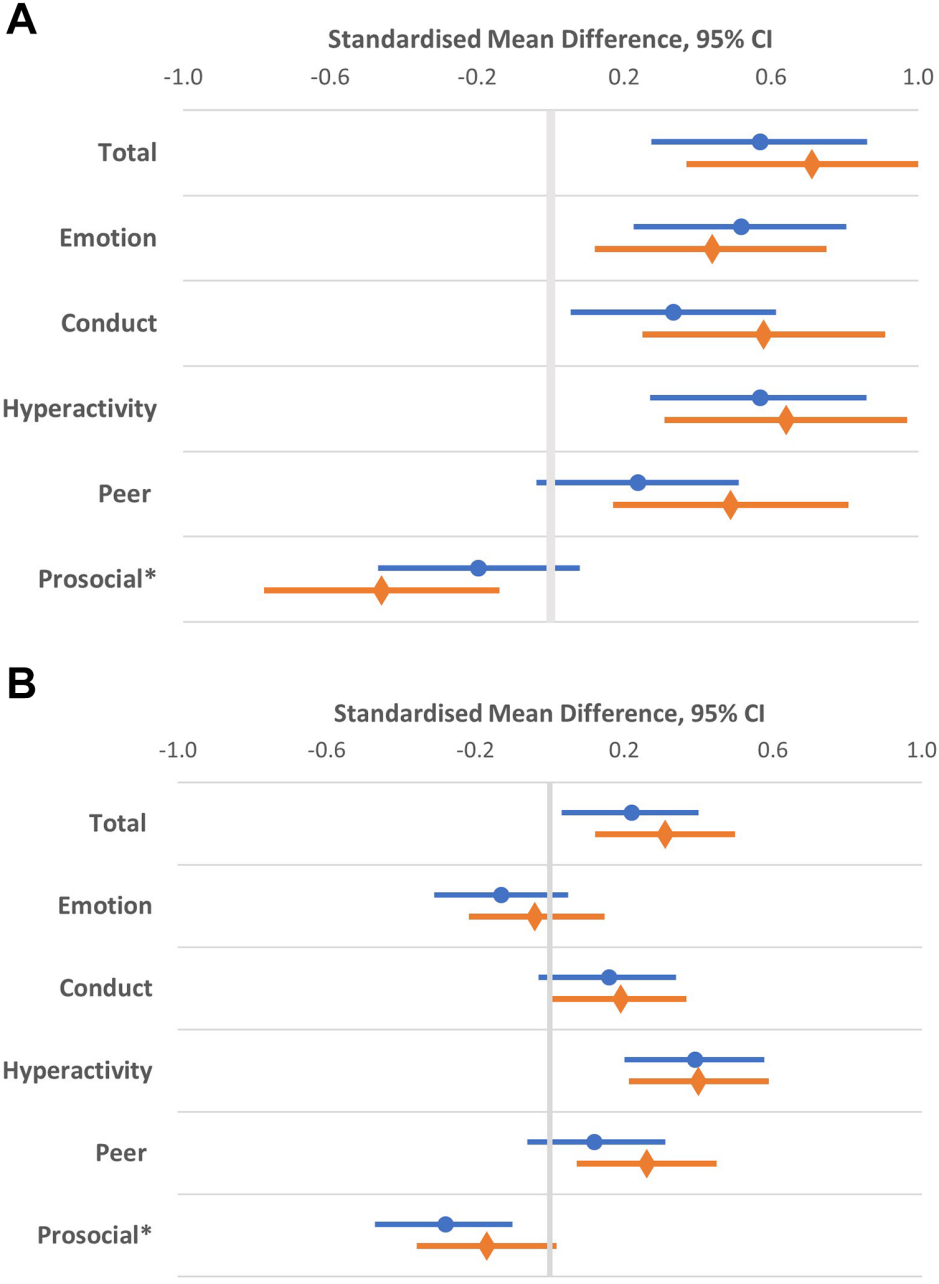
Compared to Australian population normative scores, children with unilateral/mild HL (•) and moderate to profound HL (◊) were reported to have higher mean differences for total and emotional/behavioral subscales according to age (**A**) 5-6 and (**B**) 7-12 years. *Lower prosocial behavior scores indicate difficulties.

In Figure 2, we demonstrate emotional/behavioral difficulties, total and the subscales mean score differences of both DHH groups, according to age groups. Emotional/behavioral difficulties total scores of children with unilateral/mild HL and moderate to profound HL were comparable (mean 9.8, SD 6.4 and mean 10.5, SD 6.5). However, younger children 5–6 years experienced more emotional/behavioral difficulties, with total scores within the top 20% (borderline) range (mean 10.1, SD 6.2 for unilateral/mild HL and mean 11.4, SD 6.9 for moderate-profound HL), while scores for children 7–12 years in both groups were within normal range (mean 9.6, SD 6.5 and mean 10.2, SD 6.3), for both DHH groups.

Compared to population normative scores, children with unilateral/mild HL and moderate to profound HL were reported to have higher emotional/behavioral difficulties scores for both age groups. Among all the subscales, hyperactivity symptoms had the highest mean scores differences compared to population normative scores, for both age and DHH groups. Table 2 estimates the standardized mean difference in HRQoL and parent distress outcome scores of children with unilateral/mild HL and moderate to profound HL compared to norms. Parents of DHH children from both groups reported poorer HRQoL for their child and higher parent distress scores compared to normative population scores (Table 2). HRQoL total and parent distress mean scores between both DHH groups were similar (74.1 (SD 18.4) vs. 72.8 (SD 17.9) and 15.5 (SD 3.6) vs. 15.0 (SD 3.7)). Poorer HRQoL in all domains and increased parent distress were also correlated with greater emotional/behavioral difficulties, of moderate effect size (r) ranging from 0.22–0.71 (*p* < 0.001), indicating that children with emotional/behavioral difficulties tended to have poorer HRQoL and lived with a parent with high levels of psychological distress.

**Table 2:** Child related HRQOL and parent distress standardized mean differences (SMD) of children with unilateral/mild bilateral HL and moderate-profound HL compared with norms.

	Norm(ref)	Unilateral/ mild HL	Effect size	*p*-value	Moderate- profound HL	Effect size	*p*-value
		Mean (SD)	(SMD) (95% CI)		Mean (SD)	(SMD) (95% CI)	
Parent distress	5.9 (4.3)	15.5 (3.6)	2.64 (2.27, 3.0)	<0.001	15.0 (3.7)	2.44 (2.08, 2.80)	<0.001
HRQOL							
Total	81.3 (15.9)	74.1 (18.4)	−0.39 (−0.54, −0.23)	<0.001	72.8 (17.9)	−0.47(−0.63, −0.31)	<0.001
Physical health	83.3 (20.0)	78.8 (23.9)	−0.18 (−0.22, −0.03)	0.022	78.4 (23.9)	−0.21(−0.36, −0.05)	0.008
Psychosocial	80.2 (15.8)	72.1 (16.6)	−0.49 (−0.65, −0.33)	<0.001	69.9 (17.6)	−0.78(−0.93, −0.59)	<0.001
Emotion	80.3 (17.0)	68.9 (18.6)	−0.61(−0.77, −0.44)	<0.001	69.7 (19.3)	−0.55(−0.71, −0.38)	<0.001
Social	82.2 (20.1)	76.3 (21.4)	−0.28(−0.43, −0.12)	<0.001	71.7 (23.1)	−0.46(−0.61, −0.30)	<0.001
School	76.9 (20.2)	71.1 (18.2)	−0.32(−0.47, −0.16)	<0.001	68.5 (18.8)	−0.45(−0.61, −0.29)	<0.001

### Aim 2: factors associated with emotional/behavioral outcomes in children with unilateral/mild HL

Table 3 illustrates the association between each potential factor and emotional/behavioral difficulties (as measured by the SDQ scores; dichotomized) in children with unilateral/mild HL. The strongest evidence of association were additional health needs, demonstrating an approximately 1.7-fold increase in odds of emotional/behavioral difficulties (OR = 1.67; 95% CI 1.29–2.17, *p* < 0.001) with every additional health need. There was weaker evidence of association with demographic characteristics and early developmental concerns, nevertheless, the association between early developmental concerns, particularly gross motor milestone and attending mainstream school with emotional/behavioral difficulties were noteworthy. To investigate the association between each developmental domain and additional health needs, correlation analysis showed that children with unilateral/mild HL and additional health needs were more likely to have early problem-solving developmental concern reported at 36 months or younger, [*r* (98): 0.30, *p* = 0.002]. No association between additional health needs and other early developmental domains were demonstrated (Communication: *r*(98):0.09, *p* = 0.36; gross motor: *r*(98): 0.09, *p* = 0.35; fine motor: *r* (98): 0.08, *p* = 0.41; social: *r*(98): 0.04, *p* = 0.70).

**Table 3:** Estimated associations (quantified as odds ratios) between child characteristics and emotional/behavioral outcomes for children with unilateral/mild HL.

	Emotional/behavioral difficulties	Odds ratio	*P* value
	(*N* = 31)	(95% CI)	
Gender, male, *n* (%)	15 (48.4)	1.08 (0.50, 2.36)	0.839
Age, 5– 6 years, *n* (%)	10 (32.3)	0.92 (0.40, 2.12)	0.842
Hearing laterality, bilateral, *n* (%)	16 (51.6)	0.91 (0.41, 2.00)	0.807
Hearing aid first fitting (*n* = 102), median, (IQR) months	24.0 (49.8)	1.01 (0.99, 1.03)	0.55
Cochlear implant first fitting (*n* = 6), median (IQR), months	87.0^a^	-	-
Type of hearing loss, sensorineural, *n* (%)	19 (61.3)	1.93 (0..85,4.39)	0.115
Hearing device, unaided, *n* (%)	13 (41.9)	0.91 (0.39, 2.13)	0.836
Communication mode, speech only, *n* (%)	25 (80.7)	4.72 (0.63, 35.12)	0.13
School, mainstream ± special unit, *n* (%)	22 (70.9)	3.30 (0.99, 11.02)	0.053
Additional health needs, *n* (%)	31 (100.0)	1.67 (1.29, 2.17)	<0.001
Early developmental concerns, *n* (%), (*n* = 100)			
Communication	4 (26.7)	0.98 (0.28,3.39)	0.975
Gross motor	5 (33.3)	3.36 (0.97, 11.70)	0.056
Fine motor	4 (26.7)	3.07 (0.81, 11.69)	0.1
Problem solving	4 (26.7)	2.73 (0.73, 10.22)	0.137
Social/Adaptive skill	4 (26.7)	3.07 (0.81, 11.69)	0.1

^a^ Numbers too small for analysis.

More than one third (39.5%) of children with unilateral/mild HL were unaided. The use of hearing devices and other audiological factors showed weak evidence of association with emotional/behavioral difficulties (Table 3). Table 4 further compared audiological factors and outcomes between children with unilateral HL and mild HL. More children with unilateral HL were unaided (65.5% vs. 17.6%) and had their first fitting with hearing aids at an older age (median age 24.0 months vs. 16.7 months) compared to children with mild HL. However, emotional/behavioral, HRQoL and parental psychological distress outcomes of unilateral and mild HL were comparable.

**Table 4:** Comparison of audiological factors and outcome scores of children with unilateral and mild hearing loss.

	Unilateral HL, *n* = 96	Mild HL, *n* = 73
Audiological characteristics		
Hearing device, *n* (%)		
Unaided	57 (65.5)	9 (17.6)
Hearing aid/s only	27 (31.0)	39 (76.5)
Cochlear implant	3 (3.4)	3 (5.9)
Age at first fitting of hearing aid		
Median (IQR), months	24.0 (47.0)	16.7 (26.3)
≤36 months, *n* (%)	20 (51.3)	53 (84.1)
Age of cochlear implantation, (*n* = 6)		
Median (IQR), months	30.0 (38.0)	41.0 (9.0)
≤36 months, *n* (%)	2 (66.7)	1 (33.3)
Outcomes, mean (SD); median (IQR)		
Emotional/behavioral Difficulties scores	9.8 (6.4); 8.0 (8.0)	9.6 (6.4); 9.0 (7.5)
*n* (%)	17 (17.7)	14 (19.2)
HRQOL scores,		
Total	73.8 (18.5); 77.2 (23.9)	74.6 (18.5); 78.8 (26.5)
Physical health	79.3 (25.3); 89.5 (31.0)	78.1 (24.9); 88.0 (34.0)
Psychosocial	71.5 (16.1); 71.7 (25.0)	72.9 (17.3); 75.0 (24.2)
Emotion	68.2 (18.4); 70.0 (25.0)	70.1 (18.9); 75.0 (28.8)
Social	76.3 (21.2); 80.0 (35.0)	76.2 (21.7); 80.0 (35.0)
School	70.1 (17.8); 75.0 (30.0)	72.5 (18.8); 75.0 (25.0)
Parent psychological distress (K6) scores	15.9 (3.6); 16.0 (4.0)	15.1 (3.7); 14.0 (5.0)

## Discussion

### Key findings

In a cross-sectional study of a large prospective cohort of DHH children, we showed that approximately one fifth of children 5–12 years old with unilateral/mild HL experienced emotional/behavioral difficulties, measured as higher mean SDQ scores compared to Australian normative population data. Compared to peers with moderate-profound HL, children with unilateral/mild HL experienced comparable rates of emotional/behavioral difficulties and similar child health related quality-of- life and levels of parental psychological distress. Children with unilateral/mild HL with additional health needs were at risk of emotional/behavioral difficulties. Early developmental concerns, other than communication milestone and attending mainstream school showed weaker evidence of association.

### Outcomes of unilateral/mild hearing loss

To our knowledge, this is one of the largest studies of DHH children demonstrating children with unilateral/mild HL experiencing comparable emotional/behavioral difficulties in comparison to peers with moderate-profound HL and greater than the normative population. Even though emotional/behavioral mean scores were within normal range, the wide standard deviations and high proportion of scores indicative of emotional/behavioral difficulties suggests high variability in emotional/behavioral outcomes among children with unilateral/mild HL. Prior studies of DHH children with more severe degrees of HL have reported similar results; however, our participants with unilateral/mild HL demonstrated emotional/behavioral difficulties of large effect sizes compared to norms (2, 7).

Our study demonstrated a high prevalence of hyperactivity symptoms among both groups of DHH children. Although a review study reported contradicting results (2), many earlier studies have demonstrated ADHD-like symptoms among DHH children (6, 33). Hyperactivity symptoms are not unexpected, as children with HL and ADHD share similar difficulties in executive function and self-regulation (34, 35). The reduction in emotional/behavioral difficulties scores observed among the older children in both DHH groups suggest that developmental gap narrows with age in response to intervention, adaptation to challenges and with maturity. These age-related changes were also observed in a 3-year follow-up longitudinal study that showed improvement in behavioral problems among a proportion of school age children with unilateral HL, highlighting the positive gains of intervention in a selected group of DHH children (19).

Emotional/behavioral outcomes were strongly correlated with child health related quality-of-life and parental distress levels. This association is consistent with prior reports, where parents of DHH children and adolescents with high externalizing and internalizing behaviors were more likely to report mental health problems and be burdened by the challenges faced (12, 36). Dammeyer et al. (2019) additionally reported that the degree of HL was not an influencing factor of the family's well-being (37). Families of children with unilateral/mild HL described different but consequential challenges compared to families of children with moderate to profound HL (37, 38). Parents reported feeling less support and empathy from the DHH community as their children with milder HL were perceived to be “Not deaf enough” and the significance of mild HL was minimized by healthcare providers (37). With the majority of children with unilateral/mild HL attending mainstream school, they would be required to fully rely on listening and speaking to communicate and experienced high expectations regarding their performances in academic, language and social skills. Furthermore, the perceived benefits of using hearing aids may be less obvious to both child and parents, hence many families may struggle with compliance and be frustrated or guilty when not able to follow through with intervention (37, 38). To address these unique challenges, further research capturing the various experiences of families with or without hearing devices may guide future recommendations and support required to optimize management of children with unilateral/mild HL.

### Factors associated with emotional behavioral outcomes

Among children with unilateral/mild HL, our study identified additional health needs as the only predictive factor of emotional/behavioral outcomes. Although a few studies have suggested maternal education level and age of diagnosis/intervention as possible factors (20, 24), we did not identify other demographic or audiological factors that were significantly associated with emotional/behavioral outcomes. Numerous studies of DHH children have shown that additional disabilities and lower cognitive skills are associated with poorer outcomes and our study has identified a similar risk factor among children with unilateral/mild HL (10, 39, 40). Our finding is further supported by Wake et al. (2006)'s study that showed excellent outcomes among selected children of slight/mild hearing loss with no additional medical illness or intellectual disabilities (23). Children with HL and additional cognitive or physical comorbidities have more challenges that would impact their early milestones and response to audiological interventions. Furthermore, families of children having additional health needs besides HL are more likely to have marital and psychological distress that affects parent-child relationship (36). With over two thirds of children with unilateral/mild HL in this study having additional health needs, the need for early medical and developmental screening is warranted regardless of the degree/laterality of HL.

Our study is the first to examine early developmental profiles and the association with later emotional/behavioral outcomes among children with unilateral/mild HL. Early developmental screening of young DHH children may identify children at risk of later cognitive and educational difficulties (41). Among the five developmental domains screened, early problem-solving development is the domain most representative of early cognition and adaptive skills, hence it was not surprising that it was the only milestone found to be associated with the presence of additional health needs. Therefore, although no association was observed with emotional/behavioral outcomes, identifying young children with problem solving developmental concerns may help recognize children at risk of additional health problems. With close to one fifth of children with unilateral/mild HL reporting developmental concerns in all domains other than communication by 36 months of age, the possibility of association between early developmental concerns, particularly gross motor milestone with emotional/behavioral difficulties should not be disregard. Several studies have described the close relationship between hearing loss and motor development, suggesting that DHH children were at higher risk of deficits in balance and fine motor skills, with 12% of young DHH children detected with early gross motor developmental delay and one fifth of school age DHH children to be less competent in gross and fine motor skills compared to typical hearing peers (9, 11, 42). Children with slight/mild HL have also been reported to have significantly poorer physical HRQoL compared to typical hearing children (33) hypothesized to be related to inner ear abnormalities or the lack of environmental exposure due to sensory deprivation. Concurrent gross motor delay among DHH children is likely to intensify emotional/behavioral difficulties as the presence of motor delay in typical hearing children have been reported to have more emotional/behavior difficulties (43). However, the relationship between motor development, hearing loss and outcomes is still poorly understood and requires more substantial evidence to warrant any recommendations.

Available evidence of the benefits of hearing assistive devices among children with unilateral/mild HL in preventing emotional/behavioral difficulties are limited and debatable. Our study showed no association between the use of hearing devices and age of amplification with emotional/behavioral outcomes. An outcome study of DHH children detected across four hearing screening systems similarly found that behavior and HRQoL to be largely unaffected by the advancement in hearing screening and expressive language continued to be lower than expected among children with mild and moderate hearing loss (13). However, other studies proposed that early age of diagnosis and amplification were associated with better social skills and psychoeducation outcomes (20, 24). Fitzpatrick et al. (2022) also found no additional behavioral problems and parenting stress among 4-year-old children with unilateral/mild HL who were identified early at median age of 4.5 months (22). Regardless, substantial evidence demonstrating clear benefits of using hearing aids to promote socio-emotional growth in children with unilateral/mild HL is unavailable. Studies of DHH children with various hearing loss severity have also shown that the use of hearing devices may not be protective of emotional/behavioral difficulties despite improvement in language abilities (6, 7, 10). Despite the possible lack of association between hearing aids and emotional/behavioral outcomes, our result should be interpreted with care due to the high probability of additional unmeasured confounding factors such as hours of aided time, quality of fitting and parental psychosocial barriers (44, 45).

Our study further described the differences in characteristics and outcomes among children with unilateral HL and mild HL. Children with unilateral HL had similar scores as children with mild HL in emotional/behavioral, HRQOL and parental distress outcomes. However, more children with unilateral HL were unaided and were first fitted with a hearing aid at a later age indicating that children with unilateral HL had less access to early intervention and support compared to children with mild HL. The differences observed highlight the possible additional challenges faced by children with unilateral HL which are yet to be explored.

### Strengths and limitations

Among the many strengths, our study has included data from a large number of children with unilateral/mild HL with and without hearing devices. These children are often under-represented in research as they are less likely to have regular healthcare appointments or appear in clinical databases. We used the SDQ, a validated measure of emotional/behavioral outcomes for the range of ages of our participants, enabling referencing of our results to population norms*.* Several limitations have also been identified. While VicCHILD is a population-based cohort, responders lived in areas of relatively less socioeconomic disadvantage compared to non-responders, and therefore results may not be generalizable to those living in more disadvantaged areas The duration of daily usage of hearing aids and level of audibility among users of hearing devices were not explored and would have provided information about the compliance of hearing aid usage particularly among unilateral/mild HL children. Likewise, information of prior usage of hearing devices and duration of use among unaided children during time of assessment may provide better understanding of its influence on socioemotional development. When analyzing the association between emotional/behavioral difficulties with different variables, we noted several odds ratio with wide confidence interval. The lack of precision may be due to the small sample size of children with unilateral/mild HL and emotional/behavioral difficulties. Larger sample sized studies may be able to explore, for example, the relationship between the accumulative effects of early developmental concerns and emotional/behavioral difficulties. Similarly, for the type of school and early developmental concerns, the result should be interpreted judiciously due to the small sample of unilateral/mild HL children not attending mainstream school and with developmental concerns; however the possibility of association with other factors should not be disregard. Single parent rated assessments used in this study to evaluate emotional/behavioral outcomes may not provide a complete perspective due to differences in child behavior and assessor priorities across different settings. However, despite differences in emotional/behavioral symptoms reported among parents and teachers, recognition of emotional/behavioral difficulties by both assessors were shown to be significantly correlated (3). Hence incorporating teacher- rated measures may not influence the result but will provide a better clinical understanding of the child's behavior throughout the day. The study strength of including DHH children of variable audiological and intervention background may also be a limitation, due to the high heterogeneity of the participants. However, the variation in the participants' characteristics reflects the real world and the different challenges faced by families of children with unilateral/mild HL. We were not able to further analyze the specifics effects of additional health needs on outcomes due to incomplete data, as additional health needs were collected only after 2020. The high reported rate of additional health needs in our sample observed, may also likely be due to the comprehensiveness of the list of medical diagnosis provided to parents. Characterizing additional health needs in future studies will provide important knowledge to accurately predict high risk DHH children and understand how they influence DHH children's emotion and behavior.

## Conclusion

This study demonstrated that children with unilateral/mild HL were just as likely as children with moderate- profound HL to experience more emotional/behavioral difficulties, poorer HRQoL and higher parental distress compared to the general population. Our study results justify the provision of early access to services and support among children with unilateral/mild HL. Early developmental screening of additional health needs is crucial to identify children with unilateral/mild hearing loss who are at risk of emotional/behavioral difficulties, as early individualized intervention may improve quality of life and parental well-being.

## Data Availability

The raw data supporting the conclusions of this article will be made available by the authors, without undue reservation.
