# Supplementary material for: Emotional behavioral outcomes of children with unilateral and mild hearing loss
Source: Front Pediatr. 2023 Oct 4;11:1209736. doi: 10.3389/fped.2023.1209736 (PMC10582705; doi:10.3389/fped.2023.1209736)
Supplement: Supplementary file 1 [file Table1.docx]

Supplementary Material

Emotional Behavioral Outcomes of Children with Unilateral and Mild Hearing Loss

# Supplementary Table 1

**Characteristics of participants (both DHH groups) and non-participants**

|  | **Participants**  (N= 339) | **Non-participants**  (N=495) |
| --- | --- | --- |
| Child/Family Characteristics  Age at diagnosis of HL (months) - mean (SD)  Gender, Male – n (%)  Maternal Education – n (%)  Year 11 or less  Year 12  Tertiary or Postgraduate  Unreported  SEIFA Disadvantage Index ꝉ- mean (SD)  Family history of HL– n (%)  English as primary language at home– n (%) | 3.0 (8.9)  183 (54.1)  30 (8.8)  92 (27.1)  129 (38.1)  88 (26.0)  1013.4 (68.3)  27 (8.0)  264 (77.9) | 3.5 (11.1)  266 (53.7)  75 (15.2)  130 (26.3)  218 (44.0)  72 (14.5)  1003.4 (SD 66.8)  38 (7.7)  334 (67.5) |
| Audiological Characteristics, n (%)  Hearing Laterality,  Unilateral  Bilateral  Unreported  Degree of HL  *Unilateral/ **Bilateral Mild HL  **Bilateral Moderate – Profound  Unreported  Type of HL, Sensorineural | 96 (28.3)  243 (71.7)  0  169 (49.9)  170 (50.1)  253 (74.6) | 144 (29.1)  334 (67.5)  17 (3.4)  247 (49.9)  217 (43.8)  31 (6.3)  342 (69.1) |

*Degree of HL in the worse ear**Degree of HL in the better hearing ear
