# Supplementary material for: Emotional behavioral outcomes of children with unilateral and mild hearing loss
Source: Front Pediatr. 2023 Oct 4;11:1209736. doi: 10.3389/fped.2023.1209736 (PMC10582705; doi:10.3389/fped.2023.1209736)
Supplement: Supplementary file 2 [file Table2.docx]

Supplementary Material

Emotional Behavioral Outcomes of Children with Unilateral and Mild Hearing Loss

# Supplementary Table 2

Emotional/ behavioral difficulties and subscales scores above cut-off in children with unilateral/mild HL (n=169) and moderate-profound HL (n=170).

|  | **Unilateral/Mild HL (N=169)** | | **Moderate-Profound HL (N=170)** | |
| --- | --- | --- | --- | --- |
|  | n | % | n | % |
| **SDQ Scores ≥ Cut-Off**  Total  Emotional  Hyperactivity  Conduct  Peer  Prosocial | 31  33  38  34  25  40 | 18.3  19.5  22.5  20.1  14.8  23.7 | 35  25  38  33  37  39 | 20.6  14.7  22.4  19.4  21.8  22.9 |

.

* Cut-off scores are based on Australia normative data, retrieved from Kremer et al, 2015 and Mellow D., 2005
