# Supplementary material for: Emotional behavioral outcomes of children with unilateral and mild hearing loss
Source: Front Pediatr. 2023 Oct 4;11:1209736. doi: 10.3389/fped.2023.1209736 (PMC10582705; doi:10.3389/fped.2023.1209736)
Supplement: Supplementary file 3 [file Table3.docx]

Supplementary Material

Emotional Behavioral Outcomes of Children with Unilateral and Mild Hearing Loss

**Supplementary table 3**

Emotional/behavioral total and subscales standardized mean differences (effect size) of children with unilateral/mild HL and moderate-profound HL compared to Australian normative population means according to age.

|  | **Normative population mean (SD)** | **Unilateral/ Mild HL**  **mean (SD)** | **Effect size**  **(95% CI)** | **p-value** | **Moderate- Profound HL**  **mean (SD)** | **Effect size**  **(95% CI)** | **p-value** |
| --- | --- | --- | --- | --- | --- | --- | --- |
| **SDQ scores**  5-6 years  Total Difficulties  Emotion  Conduct  Hyperactivity  Peer  Prosocial  7-12 years  Total Difficulties  Emotion  Conduct  Hyperactivity  Peer  Prosocial | 6.53 (4.86)  1.36 (1.62)  1.18 (1.39)  2.81 (2.23)  1.17 (1.47)  8.21 (1.82)  8.2 (6.1)  2.1 (2.0)  1.5 (1.6)  3.1 (2.4)  1.6 (1.9)  8.3 (1.7) | 10.06 (6.18)  2.44(2.09)  1.73 (1.65)  4.29 (2.61)  1.60 (1.80)  7.69 (2.63)  9.59(6.49)  1.83 (2.04)  1.78 (1.79)  4.19(2.79)  1.82(1.79)  7.70(2.13) | *0.57 (0.27;0.86)  *0.52 (0.23;0.81)  *0.33(0.05;0.61)  *0.57(0.27;0.86)  0.24 (-0.04;0.51)  -0.20 (-0.47;0.08)  *0.22(0.32;0.40)  -0.13(-0.31;0.50)  0.16(0.03;0.34)  *0.39(0.20;0.58)  0.12(0.04;0.27)  *-0.28(-0.47; -0.10) | <0.001  <0.001  0.019  <0.001  0.093  0.162  0.022  0.154  0.095  <0.001  0.185  0.003 | 11.40 (6.87)  2.29 (2.11)  2.36 (2.03)  4.67 (2.89)  2.10 (1.88)  7.12 (2.37)  10.19 (6.33  2.02 (1.99)  1.80 (1.62)  4.25 (2.87)  2.11 (1.93)  7.86 (2.52) | 0.71(0.37;1.05)  0.44 (0.12;0.75)  0.58 (0.25;0.90)  0.64 (0.31;0.97)  0.49 (0.17; 0.81)  -0.46(-0.78; -0.14)  0.31 (0.14;0.49)  -0.04 (-0.21;0.14)  0.19 (0.01;0.36)  0.40 (0.22;0.58)  0.26 (0.09;0.44)  -0.17 (-0.35;00) | <0.001  0.007  <0.001  <0.001  0.003  0.005  <0.001  0.664  0.036  <0.001  0.003  0.05 |
