# Supplementary material for: Emotional behavioral outcomes of children with unilateral and mild hearing loss
Source: Front Pediatr. 2023 Oct 4;11:1209736. doi: 10.3389/fped.2023.1209736 (PMC10582705; doi:10.3389/fped.2023.1209736)
Supplement: Supplementary file 4 [file Table4.docx]

Supplementary Material

Emotional Behavioral Outcomes of Children with Unilateral and Mild Hearing Loss

**Supplementary table 4**

Correlation between emotional/behavioral outcomes with HRQOL and parental distress.

|  | **SDQ** | | | | | |
| --- | --- | --- | --- | --- | --- | --- |
|  | **Total Difficulties** | **Emotional** | **Hyperactivity** | **Conduct** | **Peer** | **Prosocial** |
| **Parental Distress** | .354** | .242** | .277** | .223* | .291** | -0.108 |
| **HRQOL**  Total | -.682** | -.422** | -.568** | -.489** | -.549** | .515** |
| Physical Health | -.554** | -.321** | -.484** | -.381** | -.453** | .428** |
| Psychosocial | -.708** | -.466** | -.567** | -.503** | -.574** | .496** |
| Emotion | -.626** | -.563** | -.442** | -.497** | -.406** | .387** |
| Social | -.587** | -.344** | -.440** | -.400** | -.573** | .428** |
| School | -.590** | -.295** | -.569** | -.406** | -.476** | .472** |

- < 0.01 ** < 0.001
